# Thermal decomposition of selected chlorinated hydrocarbons during gas combustion in fluidized bed

**DOI:** 10.1186/1752-153X-7-2

**Published:** 2013-01-06

**Authors:** Malgorzata Olek, Jerzy Baron, Witold Zukowski

**Affiliations:** 1 Department of Thermal Engineering and Air Protection, Faculty of Environmental Engineering, Cracow University of Technology, Warszawska 24, Cracow, 31-155, Poland; 2 Department of Inorganic Chemistry and Technology, Faculty of Chemical Engineering and Technology, Cracow University of Technology, Warszawska 24, Cracow, 31-155, Poland

## Abstract

**Background:**

The process of thermal decomposition of dichloromethane (DCM) and chlorobenzene (MCB) during the combustion in an inert, bubbling fluidized bed, supported by LPG as auxiliary fuel, have been studied. The concentration profiles of C_6_H_5_CI, CH_2_Cl_2_, CO_2_, CO, NO_x_, COCl_2_, CHCl_3_, CH_3_Cl, C_2_H_2_, C_6_H_6_, CH_4_ in the flue gases were specified versus mean bed temperature.

**Results:**

The role of preheating of gaseous mixture in fluidized bed prior to its ignition inside bubbles was identified as important factor for increase the degree of conversion of DCM and MCB in low bed temperature, in comparison to similar process in the tubular reactor.

**Conclusions:**

Taking into account possible combustion mechanisms, it was identified that autoignition in bubbles rather than flame propagation between bubbles is needed to achieve complete destruction of DCM and MCB. These condition occurs above 900°C causing the degree of conversion of chlorine compounds of 92-100%.

## Background

Chlorine derivatives of aliphatic and aromatic hydrocarbons are a group of compounds produced on a large scale. These substances are persistent environmental pollutants. Their degradation in nature is slow due to the presence of covalent bonds in their molecules and also they are often xenobiotics. Combustion is one of possible methods for chlorinated hydrocarbons (CHCs) destruction. Taking into account the average bond enthalpy [1] of C-Cl (338 kJ/mol) to C-C (348 kJ/mol) and C-H (412 kJ/mol)), at the elevated temperature chlorine should be easily dissociated. The main products of the process will be stable H_2_O, CO_2_ and HCl easily separated in scrubbers.

Fluidized bed incineration is one of technology which can be used in gaseous and liquid waste disposal. Usually wastes have low calorific value and auxiliary fuel (e.g. gaseous fuel) is needed to achieve stable combustion process. It is well known that in bubbling fluidized bed, gaseous fuel can be efficiently burnt [2-8]. The process of fuel conversion in inert bed material (eg. sand) is result of radical processes occurring in the bubbles [9,10]. Combustion inside the bubbles has periodic character and are accompanied by pressure pulsations and visual effects [11-14]. Gaseous mixture fed into the fluidized bed quickly reach its temperature, and then the temperature inside the bubbles reach values of several hundred degrees higher than the temperature of the fluidized bed [14]. The effect of overheating the gas mixture inside the bubbles can be used in the processes of waste disposal when the heteroatoms are present in them. Especially when heteroatoms such as Cl or Br participating in radical reactions they may cause the inhibition of the combustion process.

The combustion of solid and gaseous fuels in a fluidized bed in the presence of halogen derivatives have been studied before, but only concentration of CO, NO, NO_2_ have been analyzed. The general consensus was that the halogens have an inhibitory effect on the combustion process [15,16]. The inhibition is due to catalytic effect of Cl radicals on the process of recombination of O, H, OH, and HO_2_ radicals. It was found that the presence of HCl inhibits the oxidation of CO to CO_2_ and decreases the formation of NO in the flue gas. This effect of halogen on CO and NO concentrations in flue gases was confirmed in other works [17-21].

As studies [13] and [14] show the combustion process of gaseous fuels in a bubbling fluidized bed in the temperature range of 300–1000°C is carried out at a certain height above the distributor. It has also been shown that one can designate two characteristic bed temperatures (T_cr.1_ and T_cr.2_), which enable distinguishing of three temperature ranges (combustion regime A, B and C). These regimes are associated with different physical mechanisms of combustion. According to [14] in regime A a mixture of gaseous fuel and air burn in flame above fluidized bed. During regime B and C it burn inside the bed in bubbles. Between T_cr.1_ and T_cr.2_, in regime B, combustion process in the bubbles is possible as a result of flame propagation between bubbles. During regime C - when the temperature of the bed is higher than the critical temperature T_cr.2_ - the burning occurs as a result of self-ignition of the fuel mixture in bubbles [13,14,22]. The composition of the flue gases strongly dependences on type of combustion regime. It have been described earlier (for methane and ethane) [13,14] and will be briefly discussed later in this work (for LPG).

The aim of present work was to determine how the addition of selected chlorinated hydrocarbons influences the fluidized bed combustion over the range 300-1000°C. This was done to recognize possibility of using the reactor with an inert fluidized bed to decompose selected chlorinated hydrocarbons. Measurements of concentration of chlorine byproduct in the flue gases, under different combustion regimes were also performed. The concentration profiles of initial, final and byproduct compounds were determined as a function of mean bed temperature. The profile of degree of DCM and MCB decomposition were compared with results obtained in tubular reactors, based on data from literature.

### The experiment

#### Materials, apparatus and analytical methods

**Figure 1:**
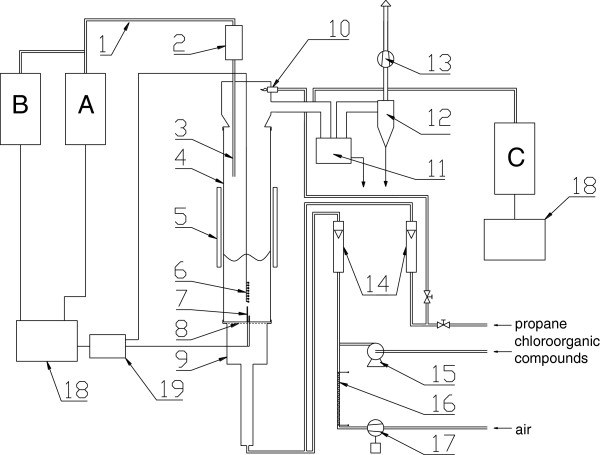
**Schematic of the lab stand.** 1- heated probe for sampling the flue gases, 2 - dust filter, 3- probe, 4- quartz pipe, 5- insulating shield, 6 – set of 8. thermocouples, 7 – two thermocouples, 8 - perforated plate, 9 - plenum chamber, 10 - pilot flame, 11 - ash trap for coarse particles, 12 – cyclone, 13- flue gases fan, 14 – rotameters, 15 - Infusion pump, 16 - electric heater, 17 - blower, for fluidizing air, 18 - computers storing chemical analyses quantities and temperature, 19 - A/D convertor for thermocouple signal, A - Horiba PG-250 analyzer, B - J.U.M. 3-200 analyzer, C - Gasmet^TM^ DX-4000 IR spectrum analyzer.

Liquefied petroleum gas (LPG) was used as hydrocarbon fuel supporting combustion process in all experiments. Dichloromethane (DCM) and chlorobenzene (MCB) - both technical grade - were chosen as chlorinated hydrocarbons. All experiments were done in lab stand, illustrated in Figure 1. The main element of it was a reactor built from a 500 mm transparent quartz tube (Figure 1, part 4, 96 mm internal diameter) resting on a 1 mm thick perforated plate Figure 1, part 8) and plenum chamber (Figure 1, part 9). The bed material was quartz sand (300 g, particle size 0.3 to 0.385 mm) with static height of 29 mm. Sand was fluidizing by a LPG and air heated to 80°C and mixed in plenum chamber. The air excess was 1.20 (±0.02). Both DCM and MCB were injected to the evaporator and then to the fluidizing air. The combustion process was initiated by a pilot flame (Figure 1, part 10) located in the freeboard. The bed temperature was regulated by a movable cylindrical insulating shield (Figure 1, part 5), which surrounded the quartz pipe and by additional fan ensured the cooling of the reaction zone. The temperature of the combustion chamber was measured using ten thermocouples (Ni-CrNi). Two of them (Figure 1, part 7) mounted from bottom, were located 20 and 50 mm above the distributor. Set of eight (Figure 1, part 6) mounted from above, were located in the axis of the reactor one above the other at a height of 5, 12, 20, 29, 40, 50, 60 and 70 mm above the distributor.

Flue gases were monitored by a set of analyzers. The most important element of the analytic set was a Gasmet^TM^ DX-4000 IR spectrum analyzer (Figure 1, part C). It utilizes Michelson interferometer to obtain spectrum of analyzed gaseous sample. After Fourier transformation of interferogram concentration of selected compounds were calculated. The gas sample was periodically analyzed to determine concentration of HCl, CHCl_3_, CH_3_Cl, CH_2_Cl_2_, COCl_2_, C_6_H_5_Cl, CH_4_, C_2_H_2_ and C_6_H_6_. Concentrations of CO_2_, CO, NO and O_2_ were continuously monitored using a Horiba PG-250 analyzer (Figure 1, part A). A J.U.M. 3-200 analyzer (Figure 1, part B) was used to monitor VOCs (volatile organic compounds). Concentrations of CO_2_ and CO were measured using non-dispersive infrared detector (NDIR), NO_x_ and O_2_ concentrations were determined using chemiluminescence (CLA) and electrochemical (EC) method respectively. The total concentration of VOCs was determined by using a flame-ionization method (FID). The accuracy of the measurement equipments for different species was as follow: CO and NO - 1 ppm, CO_2_, O_2_ - 0.1% vol., VOC - 0.1 ppm, HCl, CHCl_3_, CH_3_Cl, CH_2_Cl_2_, COCl_2_, C_6_H_5_Cl, CH_4_, C_2_H_2_ and C_6_H_6_ – 2 ppm.

### Research methodology

At the beginning of experiment run the bed of sand was fluidized by air (1.66 dm^3^/s in normal conditions), which temperature was 80°C. Then a constant stream of chloroorganic additive was injected into the air. After dosing of hydrocarbon fuel the combustion process was initiated. The bed was gradually heated from c.a. 80°C to 1000°C at rate ~1.5 K/s and for 180 s the process was carried out isothermally. Afterwards the bed was cooled down to c.a. 800°C. Then it was heated again to 1000°C. After next 180 s the process was ended by turning off the supply of chlorine additives and next by cutting off the supply of hydrocarbon fuel. Initial concentrations of DCM introduced into the reactor were as follows: 1100 ppm (DCM^1^), 2700 ppm (DCM^2^) and 5350 ppm (DCM^3^). Similar experiments were carried out using MCB to give initial concentration of 900 ppm (MCB^1^), 1800 ppm (MCB^2^) and 3650 pmm (MCB^3^), respectively. To be able to find the influence of chloroorganic addition on combustion process, a reference experiment (w/o chloroorganic compounds addition) were also carried out. In the following discussion the results for the middle concentration of DCM and MCB have been consistently ignored because the results were located between values obtained for lower and higher concentrations of additives.

## The results and discussion

### The effect of additives on the combustion process

**Figure 2:**
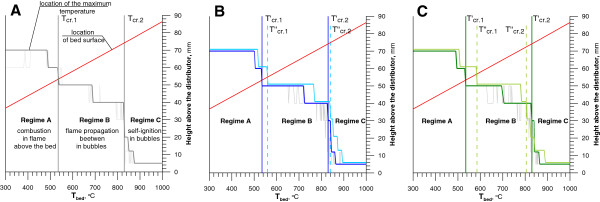
**Location of the maximum bed temperature and bed surface in relation to the average temperature of the bed. A** – experiment without the addition of chlorinated hydrocarbons, **B** – addition different streams of DCM, **C** – addition different streams of MCB (red lines– dynamic bed height; stepped lines – the position of maximum temperature: gray line – only LPG, navy blue line – 1100 ppm DCM, blue line – 5350 ppm DCM, dark green line – 900 ppm MCB, light green line – 3650 ppm MCB; continuous and dashed vertical lines indicated the critical temperature (T_cr.1_,T_cr.2_ - experiment without the addition of chlorinated hydrocarbons, T’_cr.1_, T’_cr.2_ - 1100 ppm DCM or 900 ppm MCB, T”_cr.1_, T”_cr.2_ – 5350 ppm DCM or 3650 ppm MCB).

The addition of DCM or MCB to LPG-air mixture causes changes in the process of combustion in a fluidized bed. These changes are analyzed using vertical temperature profiles registered inside the fluidized bed under different conditions. Figure 2 illustrated, as a stepped curve, the position of the highest bed temperature as a function of mean bed temperature. Red line shows location of dynamic bed surface. Taking into account previous considerations [13,14,22], the analysis of the stepped curve indicates two characteristic critical temperatures (T_cr.1_ and T_cr.2_) for each combusted mixture.

**Figure 3:**
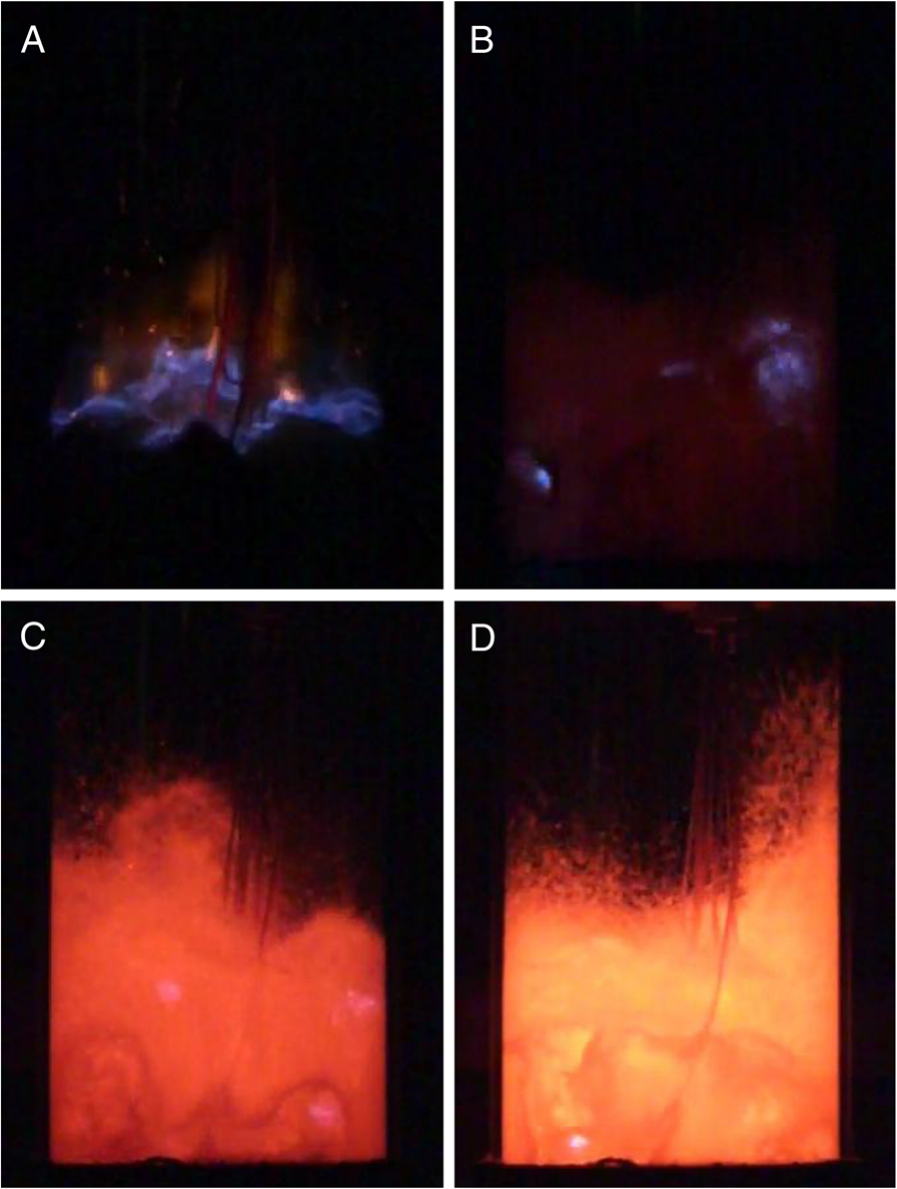
**Stages of combustion of gaseous fuel in a fluidised bed reactor (A - Combustion over the fluidized bed’s surface, Regime A, T**_**bed **_**= 200°C, the edges of the reactor is not visible, B - Combustion in a bubble leaving the fluidized bed, Regime B, T**_**bed **_**= 710°C, C - Combustion inside the fluidized bed.** Regime C, T_bed_ = 870°C, D - Combustion inside the fluidized bed, Regime C, T_bed_ = 920°C.

Figure 2A shows the reference data achieved during LPG combustion. Until the bed reaches the mean temperature of 535°C location of the combustion is above the line representing the location of the bed's surface i.e. the combustion of LPG occurred in flame above the bed (Figure 3A). Over T_cr.1_ the LPG combustion regime changed from continuous combustion above the bed to periodic combustion inside the bubbles. It occurred mostly inside the bubbles in the upper part of the bed (Figure 3B). Further temperature increase cause a slight change in location of the LPG combustion zone. Over 535°C (T_cr.1_), but below 830°C (T_cr.2_) the combustion was located inside the bed, 40-50 mm above the distributor, due to flame propagation between adjusted bubbles [13] . The dynamic height of the bed in this temperature range was between 50 – 75 mm. After exceeding 830°C (T_cr.2_) autoignition of the mixture in the bubbles occurred before it reached the bed's surface (regime C) (Figure 3C). Above 900°C the location of the most intense reactions was c.a. 5 mm above the distributor. (Figure 3D).

Adding of DCM (concentration 900 ppm – DCM^1^; Figure 2B) or MCB (concentration 1100 ppm, Figure 2C) to the reacting mixture did not change the values of critical temperatures, T’_cr.1_ and T’_cr.2_ were 535°C and 830°C respectively. Increasing the DCM concentration in the mixture at 5350 ppm and MCB to 3650 ppm increased the first critical temperature to T”_cr.1_ = 560°C (Figure 2B) and T”_cr.1_ = 585°C (Figure 2C). The second critical temperature was also changed. T”_cr.2_ = 840°C for DCM. After introducing MCB into the reactor T”_cr.2_ decreased to 800°C (Figure 2C).

The increase of the first and second critical temperature, in presence of DCM, is caused by its influence on the LPG combustion mechanism. The oxidation process in bubbling fluidized bed of inert material depends on the formation of H and OH free radicals. When Cl radicals are present at higher concentration following reactions should be taken into consideration [21]:

(1) Cl+H+M−>HCl+M

(2) $$\text{HCl}+H\left(\text{OH}\right)->H_{2}\left(H_{2}O\right)+\text{Cl}$$

In total they cause recombination of free radical concentration in reactor:

(3) $$H+H\left(\text{OH}\right)->H_{2}\left(H_{2}O\right)$$

As a result, the combustion of LPG in the presence of DCM is inhibited.

Different effects on the combustion process were observed when 3650 ppm of MCB was injected. Lowering T_cr.2_ means that not every chlorinated hydrocarbon has negative impact on the combustion process. This follows from the fact that the molar ratio C:H:Cl in the MCB (6:5:1) is different than in DCM (1:2:2).

### Products of LPG combustion in the presence of chlorinated hydrocarbons

**Figure 4:**
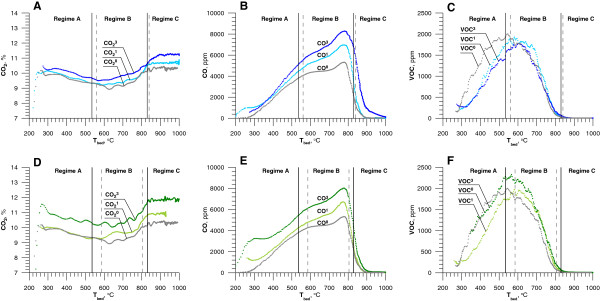
**Carbon products of combustion of LPG in an environment enriched with chlorinated hydrocarbons as a function of mean bed temperature: A, B, C – the effect of adding DCM on [CO**_**2**_**], [CO], [VOC] in flue gases (**^**1 **^**- 1100 ppm of DCM,**^**3 **^**- 5350 ppm DCM); D, E, F – the effect of adding C6H5Cl on [CO**_**2**_**], [CO], [VOC] in flue gases (**^**1 **^**- 900 ppm MCB,**^**3 **^**- 3650 ppm MCB).** For all graphs the upper index 0 - the test without the addition of chlorinated hydrocarbons. Continuous and dashed vertical lines are defined below Figure 2.

The concentrations of CO_2_, CO and VOCs at different mean bed temperature are presented in Figure 4.

Under regime A, below 200°C, the fluidization did not cause a significant turbulence on bed’s surface. LPG burns in the flame over the bed’s surface. Some of the solid particles were transported to the rare zone (freeboard) of the reactor. In that case combustion was almost completed and CO_2_ was the main carbon product. While the temperature increased, the velocity of gases and the turbulence of the fluidization increased and more solid particle were transported into the rare zone. The recombination of the radicals occurred on the surface of them. As the result a decrease of [CO_2_] and an increase of [CO] and [VOC] in the flue gases were observed (Figure 4B and C). Under regime B the combustion occurred in the large bubbles close to the bed's surface. CO_2_ concentration fell down until 630°C then increased till T_cr.2_. The concentration of VOC and CO changed, but character of the changes was different. The peak concentration of VCO reached 2000 ppm at 470 - 570°C and decays to below detection at 800°C. The inhibitive effect of the particles in fluidized bed on the processes involving radicals, which are responsible for increase of the CO concentration is clearly observed in B regime. While bed temperature increase, instead of the expected decrease [CO], it rises to maximum concentration of 5000 ppm at 780°C. Then, at temperature near T_cr.2_ [CO] fell rapidly. This was caused by the change of the mechanism controlling the combustion at the transition between B and C regimes. Above T_cr.2_ spontaneous combustion inside the bubbles takes place, in a homogenous phase without solid particles present. There was no quenching effect from recombining radical adsorbed on the sand surface. Combustion in regime C is characterized by low CO and VCO concentration. Almost entire carbon was converted to CO_2_ (Figure 4A).

After introducing DCM (Figure 4A, B and C) or MCB (Figure 4D, E and F) to the inlet mixture changes of [CO_2_, [CO] and [VOC] retained its character. Increase of the bed temperature was accompanied by the decrease of [CO_2_ till 630°C. Under regime B, above 630°C [CO_2_ gradually increased, reaching a constant value after exceeding T_cr.2_. The CO concentration increased according with temperature increased under regimes A and B. The conversion CO to CO_2_ rapidly accelerated under regime B near to T_cr.2_. During the second part of regime B the highest concentrations of CO were 6950 ppm, 7900 ppm and 8300 ppm corresponding with the concentration of DCM in inlet streams: 1100 ppm, 2700 ppm and 5350 ppm. CO concentrations resulting from the same amounts of MCB were 6725 ppm, 6980 ppm and 8035 ppm (Figure 4E). When LPG was combusted in the presence of DCM (which introduced two chlorine atoms per each additional carbon atom in the reaction zone) the CO concentration was higher. Presence of Cl free radicals and HCl has influence on the CO oxidation. The main reaction oxidizing CO to CO_2_ is [23]:

(4) $$\text{CO}+\text{OH}->{\text{CO}}_{2}+H$$

Important way of hydroxyl radical formation is by H radical via [15]:

(5) $$H+O_{2}->\text{OH}+O$$

When chlorine compound is added to the combustible mixture, Cl and HCl, compete with CO for H and OH radicals (1-2 vs. 4-5). It leads to inhibition of CO oxidation. Analyzing the changes of CO concentration (CO^1^, CO^3^ lines, Figure 4B and E) it can be determined that this inhibition mechanism is significant under regime A and B. Under regime C, at higher temperature the concentration of OH is increased and catalytic processes involving chlorine is negligible.

In the middle of the regime B the efficiency of CO_2_ (CO_2_^1^ line, Figure 4A and D) production increases, compared to experiment without chlorine additive. The mechanism reducing a negative effect of HCl on the formation of radicals substantial in carbon monoxide oxidation have been identified by Mueller et al [24]:

(6) $$\text{HCl}+\text{OH}->H_{2}O+\text{Cl}$$

(7) $$\text{Cl}+{\text{HO}}_{2}->\text{HCl}+O_{2}$$

(8) $$\text{CO}+\text{OH}->{\text{CO}}_{2}+H$$

(9) $$H+O_{2}->\text{OH}+O$$

(10) $$H_{2}O+O->\text{OH}+\text{OH}$$

In total:

(11) $$\text{CO}+{\text{HO}}_{2}->{\text{CO}}_{2}+\text{OH}$$

Under regime A when DCM or MCB was added, VOCs concentration was lower, in comparison to LPG combustion. This change indicates that below T_cr.1_, there is possibility of reaction between chlorinated byproducts and fuel which accelerates decomposition of the latter. Maximum of [VOC] was observed between regime A and B when LPG alone was burnt. Addition of CHCs shifts VOC peak slightly to higher temperature. Near T_cr.2_ VOC was completely destroyed.

### Chloroorganic compounds of the thermal decomposition of DCM

The temperature of the fluidized bed is a factor influencing the concentrations of toxic by-products resulting from incomplete combustion of DCM.

**Figure 5:**
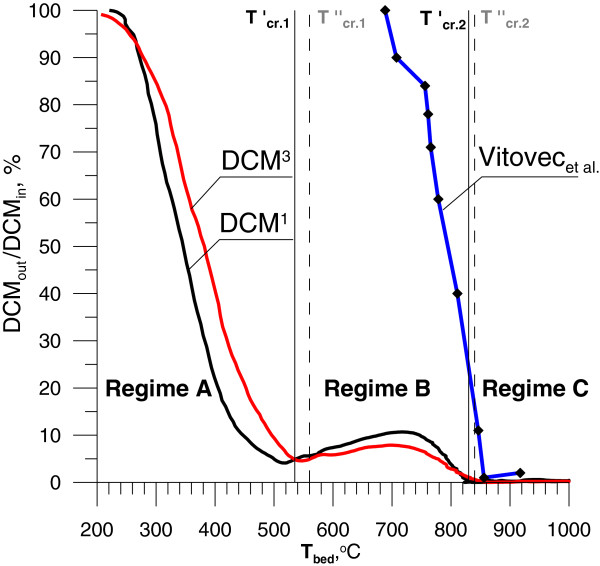
**Degree of decomposition of CH**_**2**_**Cl**_**2 **_**for different temperature (DCM**_**in **_**and DCM**_**out **_**are the inlet and outlet concentration of dichloromethane respectively).**

Degree of DCM decomposition versus mean bed temperature is shown in Figure 5. The onset of DCM’s degradation was observed at 230°C. Under regime A as the mean bed temperature increases the concentration of DCM in the combustion by-products gradually decreased. When the bed’s temperature reached T_cr.1_ the concentration of DCM in the flue gases was c.a. 4% of its initial value. Above T_cr.1_ DCM concentration was slightly increasing to reach a maximum of 8 – 10% of its initial value at 700°C. Further increase of the mean bed temperature decreases concentration of DCM. Over T_cr.2_ DCM was completely destroyed. Similar experiments carried out in the tubular reactor showed that beginning of degradation was observed at higher temperature 680°C Vitovec et al. [25]. Moreover, 50% of decomposition have been reached at 830°C compared to 350-400°C for our work. After a bubble is formed in the bed, the gaseous mixture is preheated to the temperature of the fluidized bed, and then, after delay needed to ignition the temperature rise further. It was calculated that the difference between temperature inside the bubble and mean temperature of emulsion phase in the fluidized bed is 400-500 K [22]. This effect is responsible for lowering of temperature of decomposition onset in fluidized bed in comparison with the tubular reactor.

**Figure 6:**
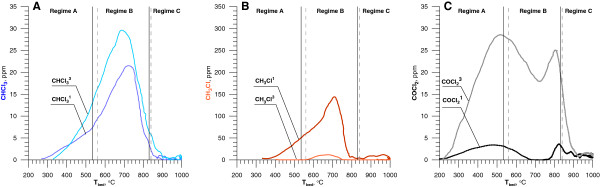
**Chlorinated byproducts of DCM as a function of the mean bed temperature (**^**1 **^**- 1100 ppm of DCM,**^**3 **^**– 5350 ppm DCM).**

Figure 6 illustrated the concentration of three chlorinated byproducts as a function of the mean bed temperature, when 1100 or 5350 ppm of DCM were added into the LPG-air mixture. Formation of chloroform started at lower temperature (280°C) than chloromethane (340°C). Concentration of both substances above these temperatures increased to reach maximum in the regime B. CHCl_3_ concentration was increasing and CH_3_Cl concentration was decreasing as the DCM stream increased. Under regime A increase of T_bed_ caused significant rise in the concentration of COCl_2_. Concentration of phosgene reached a maximum close to the first and second critical temperature at c.a. 500°C and 820°C. Between these temperatures, when the bed's temperature exceeded 740°C, the phosgene concentration temporarily dropped. This decrease of phosgene concentration was observed in regime B as CHCl_3_ and CH_3_Cl concentrations increased. Under regime C, when T_bed_ exceeded 900°C, the concentration of chlorinated byproduct drop near to zero. Above 900°C the main product of oxidation of DCM was hydrogen chloride. Introducing dichloromethane into the reactor with concentrations 1100 ppm, 2700 ppm and 5350 ppm caused an increase of hydrogen chloride concentration to 2200 ppm, 5400 ppm and 10700 ppm respectively.

In a homogenous combustion above the fluidized bed or inside the bubbles a significant role in oxidation of DCM play H radicals created during burning of hydrocarbon fuel. Initial attack on DCM can be started via reactions with H (12), Cl, O and OH radicals or oxygen particles (13)[25]:

(12) $${\text{CH}}_{2}{\text{Cl}}_{2}+H->{\text{CH}}_{2}\text{Cl}+\text{HCl}$$

(13) $$\begin{array}{l} {\text{CH}}_{2}{\text{Cl}}_{2}+\left(O_{2},\text{OH},O,\text{Cl}\right)-\\ \;>{\text{CHCl}}_{2}+\left({\text{HO}}_{2},H_{2}O,\text{OH},\text{HCl}\right) \end{array}$$

Under fuel-lean condition rather CHCl_2_ radicals will be created than CH_2_Cl [26].

According to Chi [27] the created CHCl_2_ radical reacts with radical of a chlorine to form chloroform:

(14) $$\text{CHCl}+\text{Cl}+M->{\text{CHCl}}_{3}+M$$

The observed increase of the DCM concentration when the bed's temperature was increased from 550°C to 700°C may be the result of one of the following reactions:

(15) $${\text{CH}}_{2}\text{Cl}+\text{Cl}+M->{\text{CH}}_{2}{\text{Cl}}_{2}+M$$

When methane is present in the reaction zone (e.g. it's formed as a transient product of LPG combustion) the following reaction is possible:

(16) $${\text{CH}}_{4}+\text{Cl}->\text{HCl}+{\text{CH}}_{3}$$

The methyl radicals can recombine with chlorine to form methyl chloride:

(17) $${\text{CH}}_{3}+\text{Cl}+M->{\text{CH}}_{3}\text{Cl}+M$$

In the second mechanism of CH_3_Cl formation an important role play CH_2_Cl radicals formed in reaction (12) [28]:

(18) $${\text{CH}}_{2}\text{Cl}+H_{2}->{\text{CH}}_{3}\text{Cl}+H$$

The decrease in methyl chloride concentration along with the increase in DCM concentration may indicate that reaction (18) (limited by CH_2_Cl radical concentration) has a dominating role in the fluidized bed. That radical takes part in recreating DCM during regime B according to reactions (15) (Figure 5). In temperature above 900°C both CHCl_3_ and CH_3_Cl react completely.

To create phosgene during DCM decomposition structure of two chlorine atoms bound to carbon atom is needed [25]. Phosgene formation can be initiated by chloroform dissociation [29] as follows:

(19) $${\text{CHCl}}_{3}->{\text{CCl}}_{2}+\text{HCl}$$

The CCl_2_ radical can be oxidized to phosgene:

(20) $${\text{CCl}}_{2}+O_{2}->{\text{COCl}}_{2}+O$$

COCl_2_ can also be formed during DCM destruction according to the proposed mechanism:

(21) $${\text{CH}}_{2}{\text{Cl}}_{2}+\text{OH}->{\text{CHCl}}_{2}+H_{2}O$$

(22) $${\text{CHCl}}_{2}+\text{OH}->{\text{CCl}}_{2}+H_{2}O$$

(23) $${\text{CCl}}_{2}+O_{2}->{\text{COCl}}_{2}+O$$

Phosgene can be further transformed to CO:

(24) $${\text{COCl}}_{2}+X->\text{COCl}+\text{XCl}$$

(25) $${\text{COCl}}_{2}+X->\text{COCl}+\text{XCl}$$

(26) COCl+M−>CO+Cl+M

where X = Cl, H, O, OH.

Above 900°C phosgene concentration was too small to be measured by the apparatus.

### Chloroorganic compounds of the thermal decomposition of MCB

The results support that as with the decomposition of DCM, the degree of decomposition of MCB strongly dependent on temperature of the bed.

**Figure 7:**
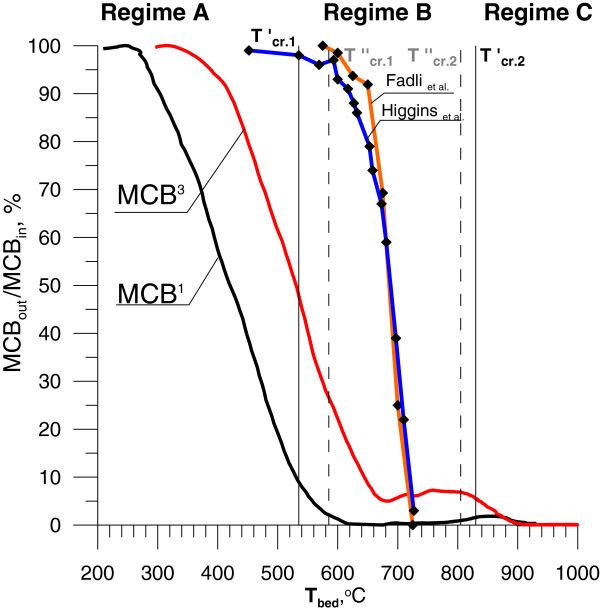
**Degree of decomposition of chlorobenzene at different temperature (**^**1 **^**- 900 ppm of MCB,**^**3 **^**– 3650 ppm MCB, MCB**_**in **_**and MCB**_**out **_**are the inlet and outlet concentration of chlorobenzene respectively).**

Figure 7 presents the decay of MCB versus the mean bed temperature, when different amount of MCB was injected into LPG-air mixture. MCB decomposition started at c.a. 300°C and proceeded slower than DCM decomposition. DCM reaching 50% decomposition at 400°C compared to 540°C for MCB (Figures 5 and 7). During regime B at 680°C the concentration of MCB reached c.a. 5% of the initial value (190 ppm). Afterwards concentration of MCB was increased to 270 ppm at 760°C. Over 900°C all MCB was destroyed. Comparison of our results to [30] Higgins et al. or [31] Fadli et al. studies, the total decomposition in the FBC occurs at higher temperatures than during thermal oxidation in flow reactor, but process begins at lower temperature. Again, the values of T_cr.1_ and T_cr.2_ are decisive factors on degree of the MCB conversion.

Below 825°C the following reactions initiating the MCB decomposition are possible [31]:

Separation of chlorine and the formation of the phenol radical:

(27) $$C_{6}H_{5}\text{Cl}->\text{Cl}+C_{6}H_{5}\left(\text{cy}\right)$$

The rupture of bonds between carbon and hydrogen:

(28) $$C_{6}H_{5}\text{Cl}->H+C_{6}H_{4}\text{Cl}$$

The breakdown of benzene ring and formation of ethyne and vinyl chloride:

(29) $$C_{6}H_{5}\text{Cl}->C_{6}H_{5}\mathrm{Cl}\left(l\right)->C_{2}\text{HCl}+{2C}_{2}H_{2}$$

Abstraction of the hydrogen or chlorine by an oxygen particle:

(30) $$C_{6}H_{5}\text{Cl}+O_{2}->C_{6}H_{4}\text{Cl}+{\text{HO}}_{2}$$

(31) $$C_{6}H_{5}\text{Cl}+O_{2}->C_{6}H_{5}+{\text{ClO}}_{2}$$

**Figure 8:**
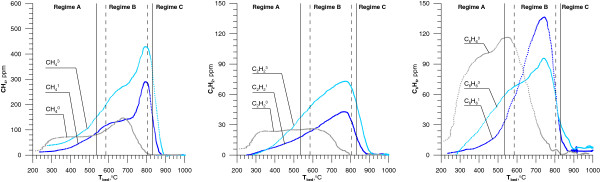
**The effect of adding MCB on [C**_**2**_**H**_**2**_**], [CH**_**4**_**], [C**_**6**_**H**_**6**_**] in flue gases (**^**0 **^**- 0 ppm MCB**^**1 **^**- 900 ppm MCB,**^**3 **^**- 3650 ppm MCB).** Continuous and dashed vertical lines are defined below Figure 2.

The perceivable products of incomplete combustion of hydrocarbons under regime B were methane and ethyne (Figure 8), which confirms the results observed by [31] Fadli et al.

From reaction 27 phenyl radicals can produce ethyne via:

(32) $$C_{6}H_{5}\left(\text{cy}\right)->C_{6}H_{5}\left(I\right)$$

(33) $$C_{6}H_{5}\left(l\right)->C_{4}H_{3}+C_{2}H_{2}$$

The reaction of methane production can involve methyl radicals, which come from propane combustion, as follows:

(34) $${\text{CH}}_{3}+C_{6}H_{5}\left(\text{cy}\right)->C_{6}H_{5}\left(I\right)$$

Introduction MCB to the system not caused a significant increase in the concentration of benzene, but shifted its maximum concentration to the higher temperature.

During the combustion of LPG with MCB the main gas product containing chlorine was hydrogen chloride. Over 900°C the conversion factor of chlorine in MCB to hydrogen chloride was 92-100%.

**Figure 9:**
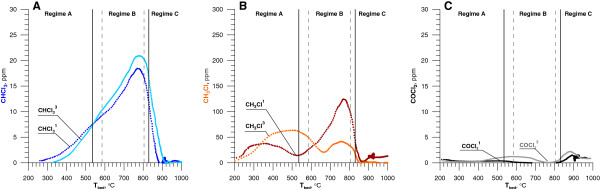
**Chlorinated byproducts of C**_**6**_**H**_**5**_**Cl decomposition as a function of the mean bed temperature (**^**1 **^**- 900 ppm MCB,**^**3 **^**- 3650 ppm MCB).** Continuous and dashed vertical lines are defined below Figure 2.

Regime B is characterized by the occurrence of the highest concentrations of products from incomplete MCB decomposition (Figure 9). In received results, the most interesting is the low concentration of phosgene. Phosgene concentration was below detection limit despite the presence of chloroform (which can be the precursor of phosgene) in the reaction zone at concentration similar to that for DCM decomposition. This means that in the conditions created in the fluidized bed the rate of the reaction (19-20) is too slow to obtain comparable amounts of phosgene during degradation of MCB. This also shows that the presence of phosgene during DCM degradation is a result of reactions (21-23). Above 900°C, under regime C, concentrations of chloride containing products of incomplete combustion are too little to be determined.

### Influence of presence of chlorine compounds on NO_x_ formation

During combustion of fuels in the air nitrogen oxides are formed by oxidation of N_2_ or nitrogen fuel. The first process runs according to the Zeldovich mechanism [32], which is important when the temperature of the reagents exceeds 1400°C, or prompt mechanism, at lower temperature [16,33]. In the latter mechanism crucial role play CH_x_ forming HCN, CN, NH and hence NO_x_.

**Figure 10:**
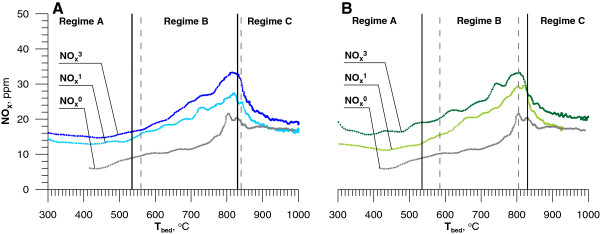
**A – Effect of adding DCM on [NO**_**x**_**] in flue gases(**^**1 **^**- 1100 ppm DCM,**^**3 **^**- 5350 ppm DCM).B** – Effect of adding C_6_H_5_Cl on [NO_x_] in flue gases (^1^ - 900 ppm MCB, ^3^ - 3650 ppm MCB) ^0^ - 0 mL/s DCM or MCB.

As Figure 10 shows NO_x_ concentration was higher when the combustion took place in the presence of chloroorganic compounds (Figure 10). The change was the result of a presence of additional pool of radicals that form quickly from CHCs even at relatively low temperature. These radicals begin a chain reaction leading to the increase concentration of CH, CH_2_ radicals and act to promote the formation of nitrogen oxides from N_2_.

## Conclusions

Thermal decomposition of chlorinated hydrocarbons during its combustion in mixture with supporting gaseous fuel, in the condition created by fluidized bed of sand, takes place in bubbles floating inside a bed’s dense phase. In relation to combustion in the tubular reactor, process of their decomposition occurs in a relatively lower temperature. This is due to specific role of the emulsion phase of fluidized bed. By contact with this phase mixture of utilized compound with air and supporting fuel are preheated to the temperature of the fluidized bed. After this preheating, the gaseous mixture (in the bubbles) ignited. When heat is released, temperature of the mixture in the bubbles increased. Due to the limited heat transfer between gases in the bubbles and the bed material, gases can reach maximum value of 1300°C or more while temperature of bed material is e.g. 850°C [14,22]. It means that the effective temperature of the degradation of the pollutant is few hundred degrees higher than the temperature measured in the reactor. What is more, intensive mixing in the bed causes the alignment of the fluidized bed temperature. This ensures appropriate conditions for complete conversion of the reactants. Combustion of hydrocarbon (supporting) fuel in such an environment ensures stable conditions and is a source of H and OH radicals, which are necessary for disintegration of chloroorganic substances. On the other hand, adding to the fuel chlorine compounds influences the combustion process which leads to some changes of the values of critical temperatures T_cr.1_ and T_cr.2_. These small shifts do not make impossible to carry on thermal degradation of given additives. It was confirmed that phosgene can be formed only if, the oxidized substance is able to create a structure of two chlorine atoms bound to carbon atom. Therefore, in the case of chlorobenzene, the concentration of phosgene in the whole temperature range, was below detection limit, while during the oxidation of DCM reached a value 11 times higher, but only if the bed temperature is lower than 900°C .

If the main efficiency criterion is the minimization of CO and NO_x_ concentration and preventing the formation of intermediate species from DCM or MCB oxidation, then it is preferable to carry out the process during regime C of the combustion in the fluidized bed. The optimum range for the process is limited by the minimum temperature of 900°C and the maximum temperature of the bed’s material. When the material used in the bed is SiO_2_ the temperature should not be higher than 1100°C. In a laboratory-scale reactor it has been found that even if the reactor has no automatic temperature regulation it is possible to have the temperature variation range at ±10 K. In a larger scale installation, which includes an automatic temperature regulation system, it is achievable to get the designated temperature range.

## 

Notice: Some of the theses presented in this work, were previously published in Polish in: Przemysl Chemiczny 91 (5), 2012, pp. 912-919

## Competing interests

The authors declare that they have no competing interests.

## Authors' contributions

All authors were equally involved in all stages of research work. All authors read and approved the final manuscript.
